# Eggplant Phenolamides: 2-Nonenal Scavenging and Skin Protection Against Aging Odor

**DOI:** 10.3390/molecules30102129

**Published:** 2025-05-12

**Authors:** Hye Mi Kim, Ji Hoon Kim, Je-Seung Jeon, Chul Young Kim

**Affiliations:** 1 College of Pharmacy and Institute of Pharmaceutical Science and Technology, Hanyang University ERICA, Ansan 15588, Republic of Korea; 2 Department of Herbal Crop Research, National Institute of Horticultural and Herbal Science, Rural Development Administration (RDA), Eumseong 27709, Republic of Korea

**Keywords:** eggplant, phenolamide, *N*-*trans*-feruloylputrescine, 2-nonenal, aging-odor, keratinocytes, anti-aging

## Abstract

Eggplants are high in polyphenols, making them a powerful antioxidant food that is beneficial for health and has excellent anti-aging effects. As metabolism slows down with aging, lipid peroxides are generated, with 2-nonenal being the main cause of old-age odor, which has a detrimental effect on skin keratinocytes. In this study, the 2-nonenal scavenging ability of fruits, leaves, stems, and roots of eggplant was evaluated, and the active compound was identified as *N*-*trans*-feruloylputrescine. Furthermore, we assessed whether the extracts and *N*-*trans*-feruloylputrescine showed a protective effect against skin damage induced by 2-nonenal. The antioxidant activity of the eggplant extracts was evaluated using DPPH and ABTS assays, and the fruits exhibited stronger antioxidant activity compared to the other extracts. Additionally, it was found that the ROS levels increased by 2-nonenal were significantly reduced by eggplant fruits and roots, which also inhibited lipid peroxidation. These results suggest the possibility of inhibiting the production of 2-nonenal itself. These findings suggest that eggplant extracts and the *N*-*trans*-feruloylputrescine can have a positive effect on preventing aging and maintaining skin health.

## 1. Introduction

Eggplant (*Solanum melongena* L.) is a widely consumed vegetable worldwide, primarily cultivated in subtropical and tropical regions, but it also grows well in temperate climates. The various varieties of eggplant produce fruits in different shapes, sizes, and colors, including green, purple, white, and yellow, with varying intensities [1]. The fruits of the eggplant are commonly consumed as a nutritious food ingredient, while its roots, stems, leaves, and dried fruits have been used for the treatment of various ailments in East Asia for centuries [2]. Eggplant is known for its health benefits, including anticancer [3], anti-inflammatory [4], antioxidant [5], hepatoprotective [6], antidiabetic [7], cardioprotective [8], and cholesterol-lowering properties [9]. These effects are primarily attributed to its rich bioactive compounds, particularly phenolic constituents, which exhibit strong antioxidants and anti-inflammatory activities that contribute to overall health and anti-aging effects [10]. Additionally, eggplant contains alkaloids (including amides and glycoalkaloids) and steroids, all of which are considered beneficial chemical constituents [10].

Among these compounds, phenolamides are typically regarded as the primary and characteristic constituents of eggplant [11]. They consist of at least one hydroxycinnamic acid derivative (such as caffeic, *p*-coumaric, ferulic, or sinapic acid) linked via an amide bond to an aromatic monoamine (e.g., tyramine, octopamine, dopamine, and serotonin) or aliphatic polyamine (e.g., putrescine, spermidine, and spermine) [12]. Phenolamides have been studied for their bioactive properties both in plants and in biological systems, including anti-inflammatory, antioxidant, anticancer, and neuroprotective activities [13,14,15]. However, research on their potential health benefits remains limited.

As metabolism slows down with aging, the increase in reactive oxygen species (ROS) and the decrease in antioxidant enzymes induce excessive lipid peroxide production. This promotes the oxidative decomposition of ω7-monounsaturated fatty acids, such as palmitoleic acid, in the skin surface lipids and produces 2-nonenal, an unsaturated aldehyde [16]. In addition, the concentration of ω7-monounsaturated fatty acids increases in skin surface lipids along with lipid peroxides with age [17]. Therefore, 2-nonenal acts as a characteristic body odor marker of the aging process [16]. 2-nonenal is mainly produced in the elderly, but it can also be produced in patients with chronic diseases whose metabolism is not smooth or patients with limited mobility, causing a characteristic unpleasant, oily, and grassy odor [16,18,19].

Several studies have demonstrated that polyphenols and their derivatives exhibit deodorizing properties. For instance, green tea, containing major catechins such as (–)-epigallocatechin gallate, (–)-epigallocatechin, (–)-epicatechin gallate, and gallic acid, has shown strong deodorant effects [20]. Similarly, tannins in persimmons and the condensed tannins and flavonoids in chestnut inner shell extract have also contributed significantly to deodorizing effects [21,22]. Recent research has indicated that 2-nonenal promotes apoptosis in keratinocytes and reduces the number of proliferating cells and the thickness of skin layers in a 3D epidermal model [23].

Therefore, controlling 2-nonenal production not only alleviates aging-associated body odor through deodorizing effects but also helps protect keratinocytes from its harmful biological actions. Building upon this hypothesis, our previous research demonstrated that kukoamine B derived from Lycii Radicis Cortex offers protective effects against 2-nonenal-induced skin damage [24]. In the present study, we report for the first time the novel biological activities of extracts from different parts of eggplant and the phenolamide compounds identified therein. While the skin-protective effects of phenolamides have been primarily studied in the context of tyrosinase inhibition [14], our findings reveal their additional potential in protecting the skin.

In this study, we comprehensively evaluate the antioxidant capacity of extracts from various parts of eggplant (fruits, leaves, stems, and roots) in relation to 2-nonenal, including their effects on ROS suppression and lipid peroxidation inhibition. We highlight the skin-protective efficacy of the extracts from multiple angles, identifying previously unreported physiological activities relevant to skin health. Our findings underscore the novel potential of eggplant-derived compounds, not only for their deodorizing effects through direct interaction with 2-nonenal but also for alleviating its harmful biological effects, positioning them as promising candidates for anti-aging and dermatological applications.

## 2. Results and Discussion

### 2.1. Antioxidant Activity of Eggplant Extracts

Prior to evaluating the antioxidant activity, the chemical composition of eggplant extracts was considered based on previously published studies. These reports identified 37 compounds, including 27 phenylpropanoid amides such as *N*-*trans*-caffeoylputrescine, *N*-*trans*-*p*-coumaroylputrescine, and *N*-*trans*-feruloylputrescine, that were consistently detected across different parts of eggplant [25]. In addition, eggplant is known to contain flavonoids, carbohydrates, and other secondary metabolites [25,26]. Given the comprehensiveness and consistency of these findings, no further compound characterization was conducted in this study, and these data were referenced to interpret our biological activity results.

**Figure 1:**
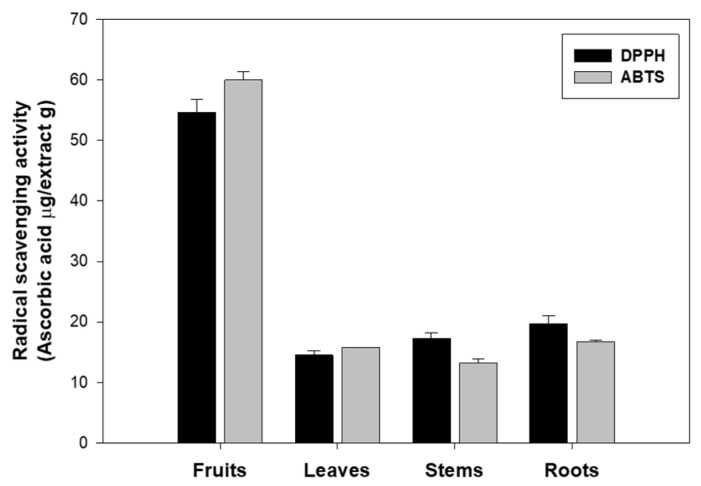
Antioxidant activity of eggplant extract evaluated by DPPH and ABTS assays. The results are expressed as ascorbic acid equivalents per gram of dry weight (μg ascorbic acid/g extract). These data are expressed as mean ± SD of three independent experiments.

Based on this background, the antioxidant activity of extracts from the fruits, leaves, stems, and roots of eggplant was evaluated using two radical scavenging assays, DPPH and ABTS. Both methods assess the free radical scavenging ability of antioxidants, and the corresponding data are shown in Figure 1. The fruits exhibited the highest antioxidant activity, with values of 54.61 μg ascorbic acid/g in the DPPH assay and 59.96 μg ascorbic acid/g in the ABTS assay. This indicates that the antioxidant substances in the fruits possess strong free radical scavenging ability in both methods.

On the other hand, the leaves showed the lowest antioxidant activity among the eggplant parts, with values of 14.51 μg ascorbic acid/g in the DPPH assay and 15.81 μg ascorbic acid/g in the ABTS assay. The antioxidant activity of the fruits was approximately 3.7-fold higher than that of the leaves in the DPPH assay and 3.8-fold higher in the ABTS assay, indicating a significantly higher radical scavenging potential in the fruits. The stems showed 17.30 μg ascorbic acid/g in the DPPH assay and 13.19 μg ascorbic acid/g in the ABTS assay, which corresponds to approximately 3.2-fold (DPPH) and 4.5-fold (ABTS) lower activity than the fruits. Similarly, the roots exhibited values of 19.73 μg ascorbic acid/g (DPPH) and 16.71 μg ascorbic acid/g (ABTS), reflecting about 2.8-fold (DPPH) and 3.6-fold (ABTS) reduced activity compared to the fruits.

These findings align with previous reports stating that eggplant ranks among the top ten vegetables with high antioxidant capacity [26,27,28,29,30]. The enhanced antioxidant activity observed in the fruits may be attributed to their higher content of phenolic compounds, including phenolamides, phenolic acids, and flavonoids, as well as anthocyanins, such as nasunin, found predominantly in the peel [3]. The variation in antioxidant activity among different parts of eggplant appears to reflect the differences in both the concentration and composition of these antioxidant phytochemicals.

However, comparisons with previous studies that analyzed the antioxidant activity of different eggplant parts reveal some inconsistencies with our findings [26,31,32]. These discrepancies may be due to differences in drying and extraction methods, cultivars used, and the specific plant parts analyzed. Notably, in our study, the eggplant fruits were not separated into peel and pulp during extraction. Since anthocyanins like nasunin are predominantly concentrated in the peel, it is likely that their presence significantly contributed to the overall antioxidant activity observed in the fruits.

### 2.2. Inhibition of Lipid Peroxidation by Eggplant Extracts

Lipid peroxidation was induced using an iron/ascorbic acid-mediated free radical generation system, based on the assumption that inhibiting the lipid peroxidation of unsaturated fatty acids could prevent the formation of 2-nonenal [33]. A previous study confirmed that metal-catalyzed oxidation increases the formation of 2-nonenal and lysine adducts from various unsaturated fatty acids, including palmitoleic acid, linoleic acid, γ-linolenic acid, and arachidonic acid [34]. This study specifically targeted arachidonic acid, a major fatty acid implicated in 2-nonenal formation, and assessed the inhibitory effects of eggplant extracts on lipid peroxidation.

The extent of lipid peroxidation was determined by measuring the residual amount of arachidonic acid using HPLC. Arachidonic acid was separated using a reverse-phase C18 column and quantified by UV detection at 210 nm. This wavelength was selected due to the strong absorbance of the conjugated diene structures in arachidonic acid, allowing for sensitive detection. In this system, oxidative degradation of arachidonic acid results in decreased peak areas in the chromatogram. Therefore, higher residual peak areas reflect stronger inhibition of peroxidation under oxidative stress conditions.

**Figure 2:**
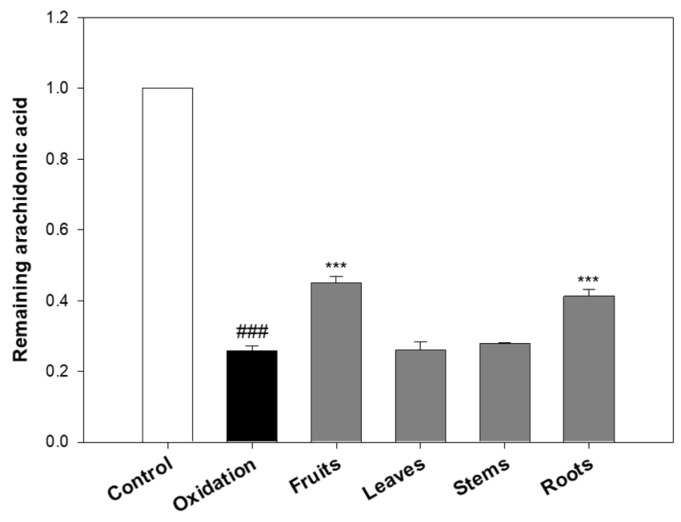
Effects of eggplant extracts on arachidonic acid reduction induced by iron/ascorbic acid-mediated lipid peroxidation. Arachidonic acid (2 mM) was subjected to lipid peroxidation induced by 250 µM FeSO_4_ and 2.5 mM ascorbic acid as oxidative triggers, with or without the addition of 2 mg/mL eggplant extracts. The control group consisted of arachidonic acid alone, without oxidative inducers. The reaction mixture was incubated at 37 °C for 1 h and then analyzed by HPLC to quantify the residual arachidonic acid. Data are expressed as the mean ± SD of 3 independent experiments. ^###^ *p* < 0.001 vs. control; *** *p* < 0.001 vs. 2-nonenal.

As shown in Figure 2, the oxidation group (treated with FeSO_4_ and ascorbic acid) significantly decreased the remaining amount of arachidonic acid to 25.91% compared to the untreated control (set as 100%), indicating that the iron/ascorbic acid system effectively induced lipid peroxidation. In contrast, treatment with eggplant extracts notably preserved arachidonic acid levels. When compared to the oxidation group, arachidonic acid levels increased by 74.16% with the fruits, 59.29% with the roots, 7.30% with the stems, and 0.97% with the leaves. These findings suggest that the fruits and roots significantly inhibited oxidative degradation, whereas the leaves and stems exhibited only marginal protective effects.

The limited efficacy of the leaves and stems is consistent with DPPH and ABTS results, which also indicated relatively low antioxidant activity in these parts. In contrast, the roots, despite their lower radical-scavenging activity in DPPH/ABTS assays, showed a strong inhibitory effect in the lipid peroxidation assay. This discrepancy suggests that certain root-derived compounds may act via specific mechanisms more effective in preventing lipid oxidation, highlighting differences between antioxidant assay systems.

Overall, these results indicate that eggplant extracts, particularly from the fruits and roots, can inhibit lipid peroxidation in vitro, potentially due to their phenolic compounds and anthocyanin content. However, as this experiment was conducted under simplified chemical conditions, its biological relevance and in vivo efficacy require further validation. Additional studies are necessary to elucidate the underlying mechanisms and confirm whether similar effects occur in cellular or physiological models of oxidative stress.

These findings suggest that eggplant extracts may play a valuable role in inhibiting lipid peroxidation. This inhibition is particularly relevant for preventing the formation of reactive aldehydes such as 2-nonenal, which contribute to oxidative stress-related skin aging. Thus, eggplant extracts hold potential as active ingredients in skin protection and anti-aging applications.

### 2.3. 2-Nonenal Scavenging Effect of Eggplant Extracts

To evaluate the scavenging ability of eggplant extracts on 2-nonenal, a key compound responsible for body odor in the elderly, extracts from the fruits, leaves, stems, and roots of eggplant were analyzed. Since 2-nonenal has low polarity and poor water solubility, it was first dissolved in ethanol before being added to the reaction system and then diluted in PBS to ensure uniform dispersion and accurate analysis. To maintain physiological conditions, the control group consisted of 2-nonenal pre-dissolved in ethanol and diluted in PBS, while the treatment groups included additional eggplant extracts under the same conditions.

The scavenging effect was assessed by quantifying the residual amount of 2-nonenal after 24 h of incubation using HPLC analysis, following previously reported methods [35]. Preliminary experiments confirmed the stability of 2-nonenal under the experimental conditions, with no significant peak shifts observed after incubation in sealed vials.

**Figure 3:**
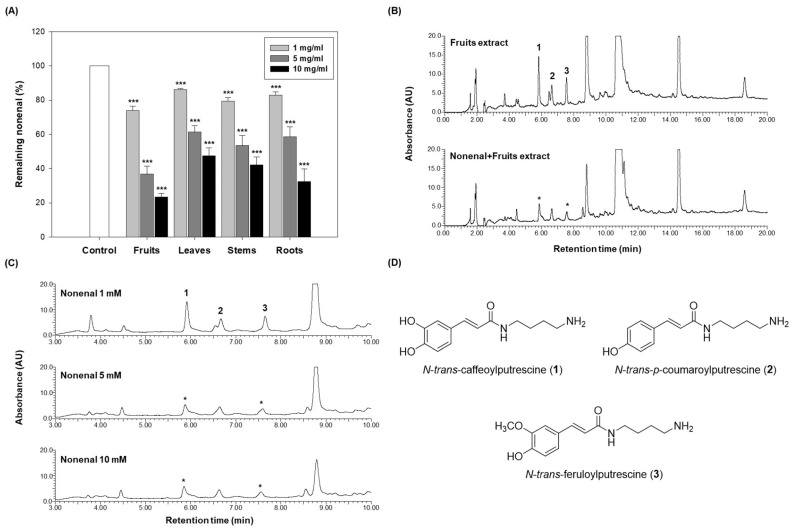
2-nonenal scavenging activity of eggplant extracts from different parts and compositional changes in fruits after reaction with 2-nonenal. (**A**) 2-nonenal (0.1 mM, in ethanol) was incubated with eggplant extracts from the fruits, leaves, stems, and roots at different concentrations (1, 5, and 10 mg/mL) in PBS at 37 °C for 24 h. After incubation, the reaction mixtures were analyzed by HPLC to quantify the residual amount of 2-nonenal. Data are presented as the mean ± SD of 3 independent experiments. *** *p* < 0.0001 vs. control. (**B**) 2-nonenal (10 mM, in ethanol) was incubated with 10 mg/mL fruits at 37 °C for 72 h. As a control, the extract was incubated with an equivalent volume of ethanol. After incubation, chromatograms were comparatively analyzed between the fruit extract alone and the reaction mixture with 2-nonenal. (**C**) The fruits (10 mg/mL) were incubated with various concentrations of 2-nonenal (1, 5, and 10 mM) at 37 °C for 24 h, and HPLC analysis was performed to assess differences in compound reduction depending on 2-nonenal concentration. Peaks marked with an asterisk (*) indicate compounds that decreased after the reaction. (**D**) Chemical structures corresponding to the peaks labeled in Figure 3**B**,**C**.

As shown in Figure 3A, all eggplant extracts reduced the residual amount of 2-nonenal in a dose-dependent manner. At a concentration of 1 mg/mL, the fruits retained 73.80% of 2-nonenal, the leaves retained 86.05%, the stems retained 79.45%, and the roots retained 83.00%. At 5 mg/mL, the residual 2-nonenal decreased in the fruits to 36.85%, in the leaves to 61.28%, in the stems to 53.44%, and in the roots to 58.53%. At the highest concentration of 10 mg/mL, the residual 2-nonenal was further reduced, with the fruits retaining 23.28%, the leaves retaining 47.43%, the stems retaining 42.07%, and the roots retaining 32.34%.

The fruits exhibited the lowest residual 2-nonenal at all concentrations, indicating the highest scavenging activity. At 1 mg/mL, the fruits retained significantly less 2-nonenal compared to the other parts. This trend continued at higher concentrations (5 mg/mL and 10 mg/mL), where the fruits consistently demonstrated the most effective reduction of residual 2-nonenal, removing approximately 80% of it at the highest concentration.

To elucidate the mechanism underlying the scavenging activity of the eggplant extract, a reaction was conducted using the fruits (10 mg/mL) and a high concentration of 2-nonenal (10 mM) to identify potential candidates responsible for this effect. To investigate chemical changes under identical conditions, an extract-only control group was prepared by adding the same volume of ethanol instead of 2-nonenal. Both the extract alone and the reaction mixture were incubated at 37 °C for 72 h. The HPLC chromatograms of the 2-nonenal-treated extract and the ethanol-treated control were then compared. As shown in Figure 3B, differences in peaks 1 and 3 were observed between the reaction mixture and the extract-only control. Furthermore, as the concentration of 2-nonenal increased, the peak intensities of compounds **1** and **3** decreased in a dose-dependent manner (Figure 3C).

These results suggest that specific compounds in the eggplant fruits may directly react with 2-nonenal, leading to the formation of new products or the degradation of active constituents, thereby contributing to its scavenging activity.

Peaks 1, 2, and 3 observed in the HPLC chromatogram were identified as *N*-*trans*-caffeoylputrescine (**1**), *N*-*trans*-*p*-coumaroylputrescine (**2**), and *N*-*trans*-feruloylputrescine (**3**), respectively, through LC-ESI-MS analysis. All three compounds were classified as phenolamides (Figure 3D). However, significant differences were observed only in compounds **1** and **3** in the chromatograms of the reactant and extract alone (Figure 3B,C). A comparison of the contents of compounds **1** and **3** in the extracts from each part revealed that compound **1** was present at a low level in the stems and roots, but at a relatively high level in the fruits and leaves, with no significant difference between the two parts (Figure S1). On the other hand, compound **3** showed the highest content in fruits, with a significant difference compared to other parts (Figure S1). Considering the correlation between these results and the scavenging activity on 2-nonenal, it is suggested that compound **3** is more likely to be the primary active ingredient rather than compound **1**. These findings support the hypothesis that compound **3** may serve as a key contributor to the observed biological effects of the eggplant extracts.

### 2.4. Identification of Active Compound and Proposed Reaction Mechanism

**Figure 4:**
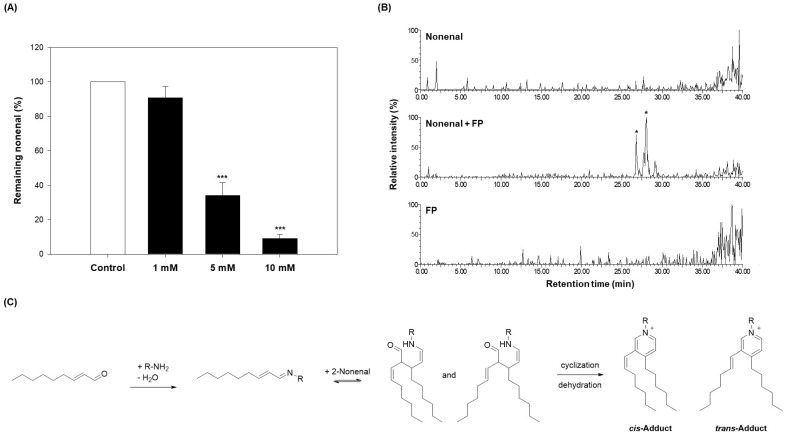
Analysis of 2-nonenal scavenging activity and proposed reaction mechanism of *N*-*trans*-feruloylputrescine (**3**). (**A**) 2-nonenal (0.1 mM, in ethanol) was incubated with *N*-*trans*-feruloylputrescine (**3**) at different concentrations (1, 5, and 10 mM) in PBS at 37 °C for 24 h. After incubation, the reaction mixtures were analyzed by HPLC to measure the residual amount of 2-nonenal. Data are presented as the mean ± SD of 3 independent experiments. *** *p* < 0.001 vs. control. (**B**) 2-nonenal (10 mM) was incubated with or without 10 mM *N*-*trans*-feruloylputrescine (**3**) at 37 °C for 72 h. As a control, *N*-*trans*-feruloylputrescine (**3**) was incubated with an equivalent volume of ethanol. After incubation, chromatographic profiles were compared between compound **3** alone and its reaction mixture with 2-nonenal. Peaks marked with an asterisk (*) indicate newly formed adducts resulting from the reaction. (**C**) Proposed mechanism of covalent adduct formation between 2-nonenal and *N*-*trans*-feruloylputrescine (**3**).

Compound **3** was selected based on its prominent peak variation in the HPLC chromatogram (Figure 3B) and its higher abundance in the fruits, which showed the strongest 2-nonenal scavenging activity. These observations suggested that compound **3** may significantly contribute to the observed bioactivity and thus warranted further investigation. To confirm that *N*-*trans*-feruloylputrescine (**3**) is an active compound, the 2-nonenal scavenging effect was evaluated. To this end, 0.1 mM of 2-nonenal was reacted with compound **3** at various concentrations (1, 5, and 10 mM) in the same manner as the scavenging effect evaluation method used for the eggplant extracts, and the residual amount was analyzed. As shown in Figure 4A, compound **3** scavenged 2-nonenal in a concentration-dependent manner. When treated with compound **3** at 1 mM, the residual 2-nonenal was 90.93%, indicating that approximately 9.07% of 2-nonenal was removed. With 5 mM treatment, the residual 2-nonenal decreased to 34.17%, showing a more significant reduction compared to the 1 mM treatment. At the highest concentration of 10 mM, compound **3** further reduced the residual 2-nonenal to 9.27%, demonstrating over 90% removal of 2-nonenal. These results confirm that compound **3** directly contributes to 2-nonenal removal in a dose-dependent manner.

From a chemical structural perspective, 2-nonenal is an *α*, *β*-unsaturated aldehyde that exhibits high reactivity toward nucleophilic functional groups. The C-3 double bond in 2-nonenal is electrophilic, making it susceptible to nucleophilic attack. Additionally, the aldehyde group at C-1 can react with an amine group to form a Schiff base [36]. Based on this reactivity, when a target compound combines with 2-nonenal, a new adduct is formed, through which 2-nonenal can be removed.

To verify this, compound **3** was reacted with 2-nonenal, and LC-ESI-MS analysis was performed. As a result, new adduct peaks were identified in the chromatogram of the reaction product, and two adduct peaks (*m*/*z* 507.6) were detected (Figure 4B and Figure S2). These results suggest that compound **3** reacts with 2-nonenal to form a new adduct, thereby contributing to its scavenging activity.

Additionally, when 2-nonenal was reacted with the fruits, LC-ESI-MS analysis was performed, confirming that compounds **1** and **3** reacted with 2-nonenal to form adducts. In particular, compound **3** produced more adducts than compound **1**, and this trend was consistent across comparisons of other parts besides the fruits (Figures S3 and S4). The amount of adduct produced in the fruits was the largest, which was consistent with the 2-nonenal scavenging activity results (Figure 3A and Figure S4). These findings suggest that compound **3** plays an important role in the 2-nonenal scavenging process and suggest that the fruits, which contain the largest amount of compound **3** and exhibit the highest scavenging activity.

Upon further investigation of the reaction mechanism between 2-nonenal and compound **3**, it was found that the amine group (-NH_2_) of compound **3** nucleophilically attacks the aldehyde group (-CHO) of 2-nonenal to form a Schiff base, followed by dehydration and cyclization to ultimately form a pyridinium derivative (Figure 4C). In this process, the imine (C=N) bond of the Schiff base can undergo geometric isomerization, resulting in the formation of *cis*- and *trans*-isomeric adducts of compound **3** with 2-nonenal. Therefore, the two adducts generated have the same molecular weight but exhibit different stereochemical configurations [34].

In previous studies, *N*-*trans*-feruloylputrescine was reported to contribute to the scavenging of 2-nonenal through synergistic effects with another active compound [24]. However, in this study, when comparing the scavenging activities of eggplant extracts from different parts, *N*-*trans*-feruloylputrescine, one of the three putrescine-derived compounds, was identified as the main active compound responsible for the strong scavenging activity observed in the fruits. Notably, *N*-*trans*-feruloylputrescine was found in the highest concentration in the fruits, which aligns with the observation that the fruits exhibited the strongest scavenging activity. This finding highlights the distinct contribution of *N*-*trans*-feruloylputrescine as the key active compound, in contrast to previous studies where it was suggested to work synergistically with another compound.

### 2.5. Protective Effects of Eggplant Extracts and N-Trans-Feruloylputrescine Against Skin Damage Induced by 2-Nonenal

In a previous study, 2-nonenal was found to decrease the viability of cultured human keratinocytes and promote apoptosis [23]. Therefore, this study aimed to evaluate the protective effects of extracts derived from different parts of the eggplant and its active compound, *N*-*trans*-feruloylputrescine (**3**), on keratinocytes damaged by 2-nonenal.

To assess cytotoxicity, keratinocytes were first treated with various concentrations (1, 5, and 10 µg/mL) of extracts from each part of the eggplant. No significant changes in cell viability were observed at any concentration (Figure S5A), indicating that the extracts themselves were nontoxic. Next, the protective effects of the extracts were evaluated by pre-treating the keratinocytes for 2 h with the extract, followed by treatment with 100 μM 2-nonenal and incubation for 24 h. The concentration of 2-nonenal (100 µM) used in this study was determined based on preliminary experiments [24]. Treatment with 150 µM 2-nonenal resulted in a sharp decrease in keratinocyte viability (~55%), which was considered too severe for a comparative evaluation of antioxidant or protective effects. In contrast, 100 µM induced moderate but measurable cellular damage (~80% viability), allowing for consistent assessment of cellular responses, such as ROS production, viability rescue, and adduct formation. Thus, 100 µM was selected as the working concentration to maintain biological relevance and experimental consistency.

**Figure 5:**
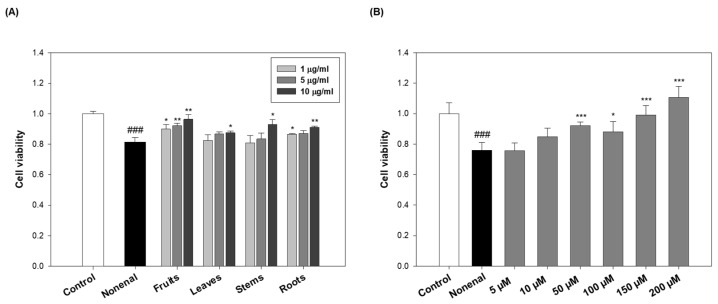
Effects of eggplant extracts and *N*-*trans*-feruloylputrescine (**3**) on 2-nonenal-induced damage in human keratinocytes. (**A**) Cell viability of HaCaT cells pre-treated with eggplant extracts (fruits, leaves, stems, and roots) at various concentrations (1, 5, and 10 µg/mL) for 2 h, followed by exposure to 2-nonenal (100 µM) for 24 h. (**B**) Cell viability of HaCaT cells pre-treated with *N*-*trans*-feruloylputrescine (**3**) at concentrations ranging from 5 to 200 µM for 2 h, followed by exposure to 2-nonenal (100 µM) for 24 h. These data are expressed as mean ± SD of 3 independent experiments. ^###^ *p* < 0.001 vs. control; * *p* < 0.05, ** *p* < 0.01 *** *p* < 0.001 vs. 2-nonenal.

As a result, cell viability decreased by approximately 20% compared to the control group after 2-nonenal treatment. However, pre-treatment with eggplant extracts from various parts effectively rescued keratinocyte viability against 2-nonenal-induced damage (Figure 5A). Among them, the fruits showed the most pronounced protective effect, improving cell viability by approximately 10–18%, depending on the concentration. Leaves, stems, and roots also conferred protective effects, although to a lesser extent compared to the fruits, with viability improvements generally ranging from about 1–14%. These results suggest that all parts of the eggplant exhibit some protective activity, but the fruits are the most potent in mitigating 2-nonenal-induced cytotoxicity.

In parallel, the protective effect of *N*-*trans*-feruloylputrescine (**3**), identified as an active compound, was evaluated. To assess its potential toxicity on cell viability, keratinocytes were treated with compound **3** at concentrations ranging from 5 to 200 μM. No cytotoxicity was observed within this range (Figure S5B). Based on prior adduct formation experiments indicating a 2:1 molar binding ratio between compound **3** and 2-nonenal, the highest test concentration was set at 200 μM (Figure 4C). Keratinocytes were pre-treated with compound **3** for 2 h prior to 2-nonenal (100 μM) exposure. Pre-treatment with compound **3** markedly improved keratinocyte viability in a dose-dependent manner (Figure 5B). Compared to the 2-nonenal-treated group, compound **3** at 10 μM increased cell viability by approximately 12%, and at higher concentrations, the rescue effect became more pronounced, reaching about a 46% increase at 200 μM. Notably, treatment with 150 μM and 200 μM of compound **3** restored cell viability to levels comparable to or even higher than the untreated control, which highlights the strong protective efficacy of compound **3** against 2-nonenal-induced damage.

These results suggest that both the eggplant extracts and compound **3** protect keratinocytes from 2-nonenal-induced cytotoxicity, primarily by direct scavenging and reduction of reactive aldehyde species. Notably, these protective effects are likely attributed to the direct interaction between the eggplant extracts/active compound **3** and 2-nonenal. Pre-treatment with the eggplant extracts or compound **3** may lead to the chemical scavenging of 2-nonenal through covalent binding, thereby reducing its availability to cause oxidative stress and apoptosis in keratinocytes.

This mechanism is supported by previous LC-MS analysis and adduct formation studies presented in this paper. Altogether, these findings highlight the potential of eggplant-derived compounds as effective skin-protective agents against aldehyde-induced aging and damage. Recent studies have shown that phenolamides play a significant role in skin protection, particularly through tyrosinase inhibition, which contributes to skin whitening effects [14]. For example, phenolamides found in bee pollen have been shown to possess anti-tyrosinase activity, while feruloylserotonin and *p*-coumaroylserotonin, derived from saffron seeds, exhibited strong melanin biosynthesis inhibition [37,38]. Additionally, compounds such as *N*-*trans*-caffeoyltyramine, *N*-dihydrocaffeoyltyramine, *N*-dihydrocoumaroyltyramine, and *N*-*trans*-feruloyltyramine have also demonstrated similar activities [39,40]. These studies suggest that phenolamides can be used as functional ingredients in cosmetics for skin whitening, anti-aging, and pigmentation inhibition.

In contrast, this study presents a new aspect of skin-related activity that has not been previously reported. Specifically, we identified that phenolamide compounds, including N-trans-feruloylputrescine, exhibit antioxidant protective effects against skin damage induced by oxidative stress, such as that caused by 2-nonenal. These compounds were found to protect against cellular toxicity and oxidative damage, introducing their novel application as antioxidative skin-protective agents in cosmetics. This finding not only expands the known role of phenolamides in tyrosinase inhibition and skin whitening but also introduces their potential in anti-aging and oxidative stress protection.

### 2.6. Effects of Eggplant Extracts on Reactive Oxygen Species (ROS) Production

Lipid peroxidation is a major feature of oxidative stress, and lipid peroxidation-derived aldehydes, such as 2-nonenal, can induce mitochondrial damage, inflammatory activation, and inhibition of the antioxidant system due to their high reactivity and toxicity. These effects can promote cell damage, apoptosis, and inflammatory responses. Accordingly, these reactive aldehydes are used as markers of oxidative stress and are associated with the development of various age-related diseases [41]. On the other hand, antioxidants regulate oxidative stress by removing reactive oxygen species (ROS) or inhibiting oxidation reactions within cells, exhibiting effects such as free radical scavenging and ROS production inhibition [42].

Previous studies have shown that eggplant extracts possess antioxidant activity and have protective effects against keratinocyte damage induced by 2-nonenal (Figure 1 and Figure 5A). Therefore, in this study, we evaluated whether eggplant extracts could inhibit oxidative stress induced by 2-nonenal.

**Figure 6:**
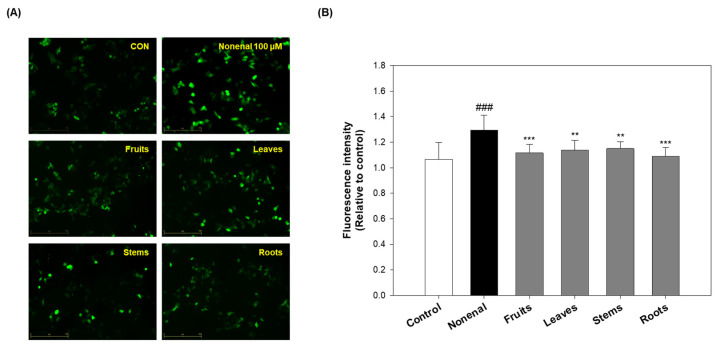
Effects of eggplant extracts on ROS production in 2-nonenal-induced damage in human keratinocytes. (**A**) Cellular ROS production was analyzed using the CM-H2DCFDA dye and observed under a fluorescence microscope (scale bar = 250 µm). HaCaT cells were pre-treated with eggplant extracts (10 µg/mL) for 2 h, followed by exposure to 100 µM 2-nonenal for 24 h. (**B**) Quantification of ROS levels was performed using ImageJ software (version 1.48) by measuring CM-H2DCFDA-positive fluorescence intensity in at least 5 randomly selected fields per well across 3 independent experiments. Data are expressed as mean ± SD from 3 independent experiments. ^###^ *p* < 0.001 vs. control; ** *p* < 0.01, *** *p* < 0.001 vs. 2-nonenal.

Analysis using the CM-H2DCFDA probe, a widely used indicator of intracellular ROS levels, showed that ROS production was significantly increased (~20%) in the 2-nonenal-treated group compared to the control group. However, pre-treatment with eggplant extracts attenuated this ROS elevation to levels comparable to the control. Specifically, ROS levels were reduced by 13.58% in the fruits-treated group, 11.94% in the leaves group, 10.98% in the stems group, and 15.78% in the roots group relative to the 2-nonenal-only group (Figure 6). Notably, the fruits and roots exhibited slightly greater ROS inhibition compared to the leaves and stems, suggesting stronger antioxidant activity in these parts.

Although the fruits consistently showed strong antioxidants and ROS-reducing effects, the roots, despite their relatively modest antioxidant activity in prior radical scavenging assays (e.g., DPPH and ABTS), demonstrated a comparable ROS inhibitory effect in this cellular model (Figure 1). This discrepancy suggests that the roots may possess ROS-modulating activity through cellular mechanisms distinct from direct radical scavenging, such as modulating intracellular antioxidant pathways or interacting with 2-nonenal to reduce oxidative damage.

The observed differences in antioxidant activity between the fruits and roots may be linked to variations in their bioactive compound compositions, which could influence their ability to modulate ROS at the cellular level. Although a direct comparison of the active compound profiles was not performed, the biological effects observed in the cellular model are significant. This suggests that different parts of the eggplant may exert antioxidant effects through distinct pathways.

Overall, these findings indicate that eggplant extracts, particularly from fruits and roots, can alleviate oxidative stress by suppressing ROS levels in keratinocytes. This highlights their potential as active ingredients in skin-protective formulations for the prevention or treatment of skin-related disorders associated with oxidative stress.

## 3. Materials and Methods

### 3.1. Chemicals and Reagents

*trans*-2-nonenal (97%) and *N*-*trans*-feruloylputrescine were purchased from TCI (Tokyo, Japan) and BOC Sciences (Shirley, NY, USA), respectively. Ethanol for extraction, as well as HPLC-grade acetonitrile, water, and formic acid, were obtained from Daejung Chemical (Siheung, Republic of Korea), while LC-MS-grade acetonitrile and water were purchased from Merck (Darmstadt, Germany).

Dulbecco’s Modified Eagle Medium (DMEM), fetal bovine serum (FBS), and penicillin/streptomycin were supplied by Gibco Corporation (Morgan Hill, CA, USA). A cell counting kit was obtained from Dojindo (Kumamoto, Japan), and CM-H2DCFDA was purchased from Invitrogen (Carlsbad, CA, USA). Phosphate-buffered saline (PBS) was obtained from Biosesang (Seongnam, Republic of Korea). All other reagents were supplied by Sigma-Aldrich (St. Louis, MO, USA).

### 3.2. Plant Materials and Extraction

The fruits, leaves, stems, and roots of eggplant were collected from Yangyang, Gangwon-do, and identified by the corresponding author (C.Y.K.). A voucher specimen (no. HYUP-SM-001) was deposited in the Pharmacognosy Laboratory of the College of Pharmacy, Hanyang University. All samples were dried at room temperature for several days and ground into a powder. The powdered samples were then extracted with 10 volumes (*w*/*v*) of aqueous ethanol using an ultrasonic water bath at room temperature for 1 h. This extraction process was repeated 3 times. After extraction, the supernatant was filtered, evaporated under reduced pressure, and stored in a refrigerator until further use.

### 3.3. Antioxidant Activity by DPPH and ABTS Assay

The DPPH (2,2-diphenyl-1-picrylhydrazyl) assay was used to evaluate the antioxidant capacity of extracts from different parts of eggplant. First, a 1 mM DPPH solution was prepared in ethanol. Then, 100 μL of a 0.3 mM DPPH solution was added to 100 μL of each extract sample in a 96-well plate. The mixture was mixed gently and incubated in the dark at room temperature for 30 min. After incubation, the absorbance of each well was measured at 515 nm using a microplate reader. Ascorbic acid was used as a standard to determine the DPPH radical scavenging activity, with concentrations ranging from 3.9 to 62.5 μg/mL.

The ABTS (2,2′-azinobis (3-ethylbenzothiazoline-6-sulfonic acid)) assay was used to evaluate the free radical scavenging activity of eggplant extracts. Briefly, an ABTS solution (7 mM) containing potassium persulfate (2.45 mM) was prepared in water and incubated in the dark at room temperature for 16 h. The solution was then diluted with ethanol (1:24, *v*/*v*). A 20 μL sample was mixed with 200 μL of the ABTS solution in a 96-well plate and incubated at room temperature for 5 min. After incubation, the absorbance was measured at 734 nm using a microplate reader. As with the DPPH assay, ascorbic acid was used as a standard to determine the ABTS radical scavenging activity, with concentrations ranging from 2.6 to 20.5 μg/mL.

The results were presented as ascorbic acid equivalents per gram of dry extract (μg ascorbic acid/g extract).

### 3.4. Lipid Peroxidation Assay

To evaluate the inhibitory effect of eggplant extracts on lipid peroxidation, a modified in vitro assay using arachidonic acid was performed. Arachidonic acid (2 mM) was dissolved in an equimolar amount of NaOH to ensure complete solubilization. Lipid peroxidation was initiated by adding 250 μM FeSO_4_ and 2.5 mM ascorbic acid. The experimental groups were as follows: (1) control group: arachidonic acid alone; (2) oxidation group: arachidonic acid treated with oxidative triggers (FeSO_4_ and ascorbic acid); (3) extract-only group: arachidonic acid with 2 mg/mL eggplant extract only (without oxidation inducers); and (4) sample group: arachidonic acid treated with FeSO_4_ and ascorbic acid in the presence of 2 mg/mL eggplant extract. Each reaction mixture was incubated at 37 °C for 1 h in a sealed vial. After incubation, the samples were filtered using a 0.45 μm PTFE syringe filter, and the residual arachidonic acid was quantified using HPLC analysis.

HPLC analysis was performed using a reverse-phase C18 column (Capcell pak C18 UG120, Osaka Soda Co., Ltd., Osaka, Japan, 4.6 mm I.D. × 250 mm, 5 µm) with UV detection at 210 nm. The mobile phase consisted of acetonitrile and water (78:22, *v*/*v*) with 0.1% formic acid, delivered at a flow rate of 1.0 mL/min. The remaining arachidonic acid was determined by comparing the peak areas among the experimental groups.

### 3.5. Quantification of Residual 2-Nonenal Using HPLC Analysis

Due to the volatility and poor aqueous solubility of 2-nonenal, it was first pre-dissolved in ethanol at a concentration of 1 mM and then diluted to 0.1 mM in PBS to ensure consistent dispersion in the reaction mixtures. This 2-nonenal-containing PBS solution was used for the control group. In contrast, the treatment mixtures were prepared by adding eggplant extracts dissolved in PBS and adjusted to the final concentrations to enable comparison with the control group under identical conditions.

Extracts from different parts of eggplant (1, 5, and 10 mg/mL) were incubated with 2-nonenal (0.1 mM) in PBS at 37 °C for 24 h. After incubation, the reaction mixtures were filtered through a 0.45 µm PTFE syringe filter prior to HPLC analysis using an Agilent 1260 Infinity HPLC system (Agilent Technologies, Santa Clara, CA, USA).

To confirm the presence of residual 2-nonenal, chromatography was performed using a Capcell pak C18 UG120 (4.6 mm I.D. × 250 mm, 5 µm, Osaka Soda Co., Ltd., Osaka, Japan). The mobile phase consisted of water containing 0.1% formic acid (solvent A) and acetonitrile containing 0.1% formic acid (solvent B) in a gradient mode: 0–30 min, 0–95% B; 30–40 min, 95% B. The flow rate was set at 1 mL/min, with an injection volume of 10 µL. Quantitative analysis of residual 2-nonenal was conducted by monitoring UV absorbance at 226 nm. The peak corresponding to 2-nonenal was identified by comparing the retention time and UV spectrum with that of the authentic standard (control). The percentage of residual 2-nonenal was calculated using the following equation:

$$\mathrm{Remaining}\;2\text{-}\mathrm{nonenal}\;\left(\%\right)\;=\;\frac{\mathrm{amount}\;\mathrm{of}\;2\text{-}\mathrm{nonenal}\;\mathrm{in}\;\mathrm{the}\;\mathrm{test}\;\mathrm{group}}{\mathrm{amount}\;\mathrm{of}\;2\text{-}\mathrm{nonenal}\;\mathrm{in}\;\mathrm{the}\;\mathrm{control}\;\mathrm{group}}\;\times \;100$$

All measurements were conducted in triplicate.

### 3.6. Identification of Active Compound and Its Adducts by LC-ESI-MS Analysis

To identify the active compounds responsible for 2-nonenal scavenging and elucidate their potential reaction mechanism, a modified reaction protocol was employed based on the previously described method. In this experiment, both 2-nonenal and eggplant extract were used at elevated concentrations (10 mM and 10 mg/mL, respectively), and the reaction duration was extended to enhance adduct formation.

Three experimental groups were prepared using PBS as the solvent under identical conditions: (1) a 2-nonenal-only group, treated with 10 mM 2-nonenal; (2) a reaction group, treated with 10 mM 2-nonenal and 10 mg/mL eggplant extract; and (3) an extract-only group, treated with 10 mg/mL eggplant extract and the same volume of ethanol used in the 2-nonenal group, replacing 2-nonenal, to ensure consistent solvent conditions. All reaction mixtures were incubated in sealed vials at 37 °C for 72 h to minimize volatilization and maintain a closed reaction system.

After incubation, the samples were analyzed by LC-ESI-MS using a Waters Acquity UPLC system (Waters, Milford, MA, USA) equipped with an electrospray ionization (ESI) source interfaced with an Advion expression CMS mass spectrometer (Advion, Ithaca, NY, USA). This analysis allowed for the comparison of the chemical profiles of each group and the identification of newly formed adducts, providing insight into the interaction between 2-nonenal and specific compounds present in the eggplant extract. Chromatographic separation was carried out using a Cosmosil 5C18-PAQ column (2.0 mm I.D. × 150 mm, 5 μm, Nacalai Tesque, Inc., Kyoto, Japan) at a flow rate of 0.2 mL/min. The mobile phase consisted of 0.1% formic acid in water (solvent A) and 0.1% formic acid in acetonitrile (solvent B). The following gradient elution program was applied: 0–6 min, 0–10% B; 6–10 min, 10–20% B; 10–20 min, 20–30% B; 20–23 min, 30–35% B; 23–34 min, 35–50% B; 34–37 min, 50–95% B; 37–40 min, 95% B, followed by a 15 min re-equilibration step. The LC eluent was introduced into the ESI interface, operating in positive ion mode. The ESI source was set to the following parameters: mass range of *m*/*z* 100–1200, capillary temperature of 200 °C, capillary voltage of 150 V, source voltage offset of 30 V, source voltage span of 10 V, source gas temperature of 150 °C, and an ESI voltage of 3500 V.

### 3.7. HaCaT Cells Culture

HaCaT human keratinocyte cell lines were purchased from Cytion (Eppelheim, Germany) and cultured in DMEM supplemented with 10% FBS and 1% penicillin-streptomycin at 37 °C in a humidified atmosphere of 5% CO_2_. HaCaT cells were subcultured every 2 d, passaged three times, and then used for experiments. For different applications, cells were seeded in various culture dishes. To evaluate cell viability, HaCaT cells were seeded at 3 × 10^4^ cells/well in a 48-well plate. For ROS measurement, cells were plated at a density of 1 × 10^5^ cells/well in a 12-well plate.

### 3.8. Cell Viability Assay

Cell viability was assessed using a cell counting kit-8 (CCK-8) according to the manufacturer’s instructions. Briefly, HaCaT cells were seeded in a 48-well plate and cultured for 24 h. The cells were then pre-treated with eggplant extracts (1, 5, and 10 µg/mL) or *N*-*trans*-feruloylputrescine (5–200 μM) for 2 h, followed by treatment with 2-nonenal (100 µM) for 24 h. After treatment, 20 µL of CCK-8 reagent was added to each well, and the cells were incubated for an additional 3 h at 37 °C. The absorbance was then measured at 450 nm using an Infinite M200 Pro Multimode Reader (Tecan, Männedorf, Switzerland).

### 3.9. Reactive Oxygen Species (ROS) Measurement

Intracellular ROS were measured using the fluorescent probe CM-H2DCFDA. HaCaT cells were pre-treated with eggplant extracts (10 µg/mL) for 2 h, followed by treatment with 100 µM 2-nonenal for 24 h at 37 °C. After the treatments, HaCaT cells were washed 3 times with PBS and incubated with 10 µM CM-H2DCFDA solution at 37 °C for 20 min in the dark. The stained cells were then washed twice with fresh culture medium and photographed using a fluorescence microscope (JuLI^TM^ stage, NanoEntek, Seoul, Republic of Korea). Fluorescent images were captured from at least 5 randomly selected fields per well across 3 independent experiments. The mean fluorescence intensity per field was quantified using ImageJ software (version 1.48).

## 4. Conclusions

The results of this study showed that the fruits, leaves, stems, and roots of eggplant, along with their active ingredient *N*-*trans*-feruloylputrescine, exhibited excellent 2-nonenal scavenging activity and provided protective effects against 2-nonenal-induced human keratinocyte damage. This suggests that eggplant extract and its active ingredients may play an important role in maintaining skin health during the aging process.

In addition, the study results demonstrated that eggplant extract can alleviate oxidative stress and enhance skin protection through antioxidant activity. This is achieved by removing or inhibiting the production of ROS, and the lipid peroxidation inhibition activity suggests the possibility of further enhancing skin protection and reducing the production of 2-nonenal.

Therefore, this study suggests that eggplant extract and *N*-*trans*-feruloylputrescine hold significant potential for the prevention and treatment of skin aging and related diseases, emphasizing their value as natural anti-aging and skin-protective agents. Furthermore, by identifying novel bioactive compounds, this study contributes to the expansion of research on bioactive food compounds and their promising applications in skin health and functional materials.

## Data Availability

The data supporting the findings of this study are available in the published article and its Supplementary Materials.

## Supplementary Materials

The following supporting information can be downloaded at: https://www.mdpi.com/article/10.3390/molecules30102129/s1, Figure S1: Quantification of two active compounds (**1** and **3**) in different parts of eggplant using LC-ESI-MS; Figure S2: Identification of adducts formed through the reaction between *N*-*trans*-feruloylputrescine (**3**) and 2-nonenal; Figure S3: Chromatograms of active compounds generated from the reaction of eggplant fruits and 2-nonenal; Figure S4: HPLC chromatograms of active compounds from eggplant extracts of different parts reacted with 2-nonenal; Figure S5: Cell viability of human keratinocytes treated with (A) extracts from different parts of eggplant or (B) *N*-*trans*-feruloylputrescine (**3**).
