# Supplementary material for: Eggplant Phenolamides: 2-Nonenal Scavenging and Skin Protection Against Aging Odor
Source: Molecules. 2025 May 12;30(10):2129. doi: 10.3390/molecules30102129 (PMC12114487; doi:10.3390/molecules30102129)
Supplement: Supplementary file 1 [file molecules-30-02129-s001.zip › molecules-3578964-supplementary.pdf]

Supplementary Material

Eggplant Phenolamides: 2-Nonenal Scavenging and Skin Protection Against Aging Odor

Hye Mi Kim [1], Ji Hoon Kim [1], Je-Seung Jeon [2] and Chul Young Kim [1,*]

[1] College of Pharmacy and Institute of Pharmaceutical Science and Technology, Hanyang University ERICA, Ansan 15588, Republic of Korea; hyemi586@hanyang.ac.kr (H.M.K.); gg890718@gmail.com (J.H.K.)

[2] Department of Herbal Crop Research, National Institute of Horticultural and Herbal Science, Rural Development Administration (RDA), Eumseong 27709, Republic of Korea; jsjeoncy@korea.kr

* Correspondence: chulykim@hanyang.ac.kr; Tel.: +82-31-400-5809

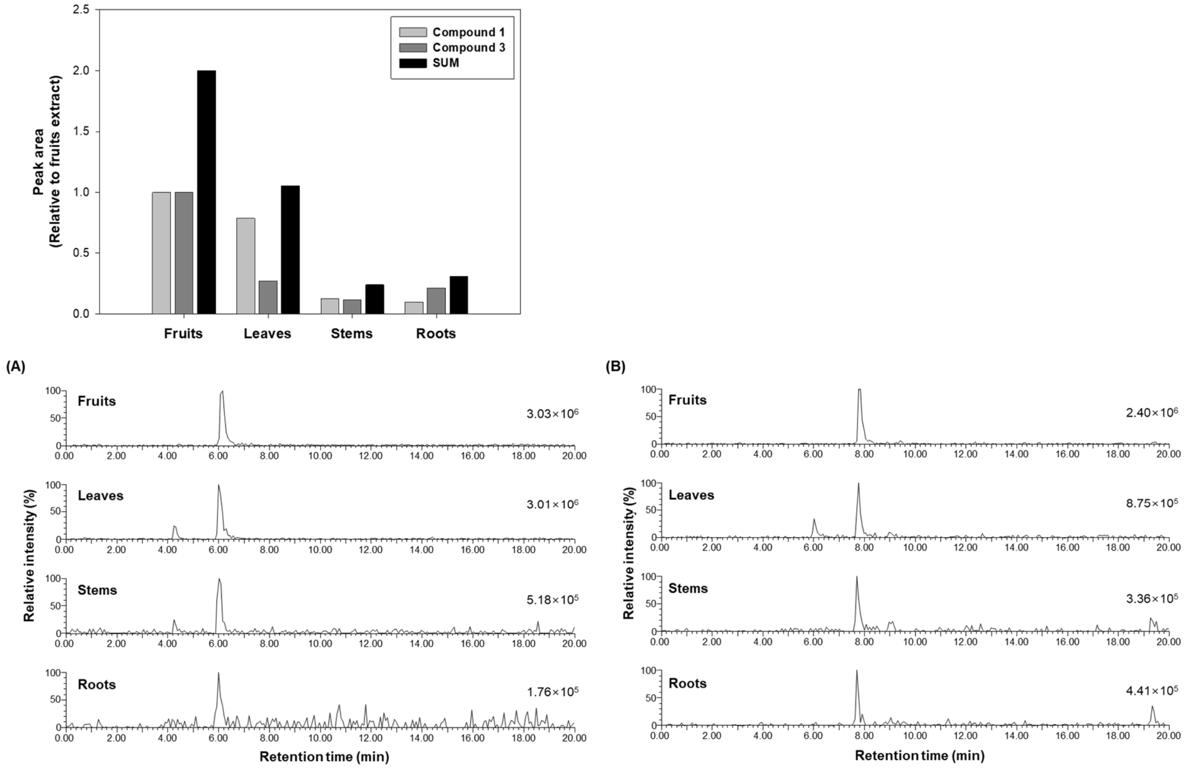

Figure S1. Quantification of two active compounds (**1** and **3**) in different parts of eggplant using LC-ESI-MS. (A) Chromatogram confirming the molecular mass (*m/z* 251) of *N-trans*-caffeoylputrescine (**1**) in extracts from different parts of eggplant. (B) Chromatogram confirming the molecular mass (*m/z* 265) of *N-trans*-feruloylputrescine (**3**) in eggplant extracts from different parts.

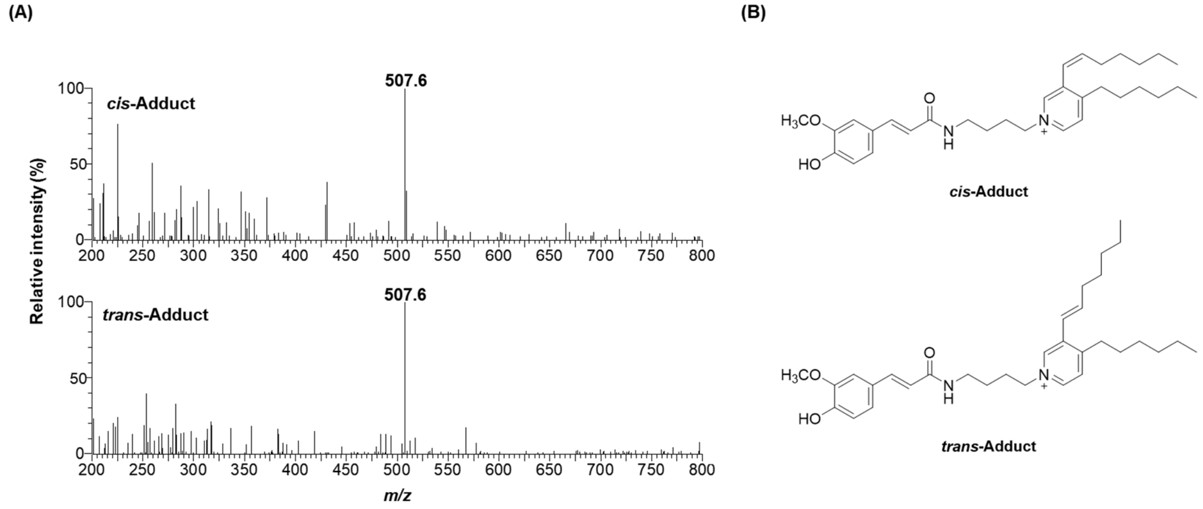

Figure S2. Identification of adducts formed through the reaction between *N-trans*-feruloylputrescine (**3**) and 2-nonenal. (A) LC-ESI-MS spectra of the adducts formed from the reaction between *N-trans*-feruloylputrescine (**3**) and 2-nonenal. (B) Proposed structures of the possible *cis*- and *trans*-adducts formed by the reaction between *N-trans*-feruloylputrescine (**3**) and 2-nonenal.

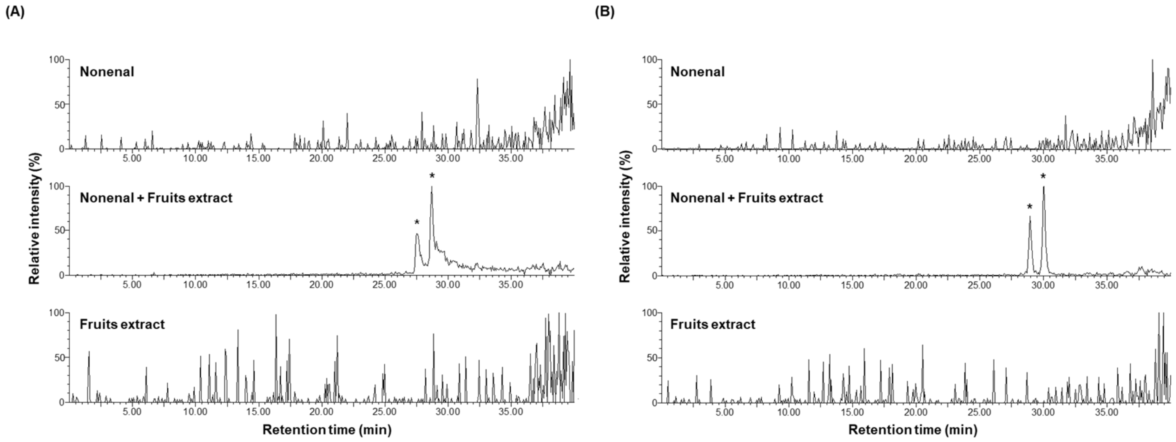

Figure S3. Chromatograms of active compounds generated from the reaction between eggplant fruits and 2-nonenal. The fruits (10 mg/mL) were incubated with 10 mM 2-nonenal at 37°C for 72 h and then analyzed using HPLC. Peaks marked with an asterisk (*) represent new product peaks formed during the reaction. (A) Chromatogram showing the product with a molecular mass of *m*/*z* 493.5, derived from the reaction of *N*-*trans*-caffeoylputrescine (**1**). (B) Chromatogram showing the product with a molecular mass of *m*/*z* 507.5, derived from the reaction of *N*-*trans*-feruloylputrescine (**3**).

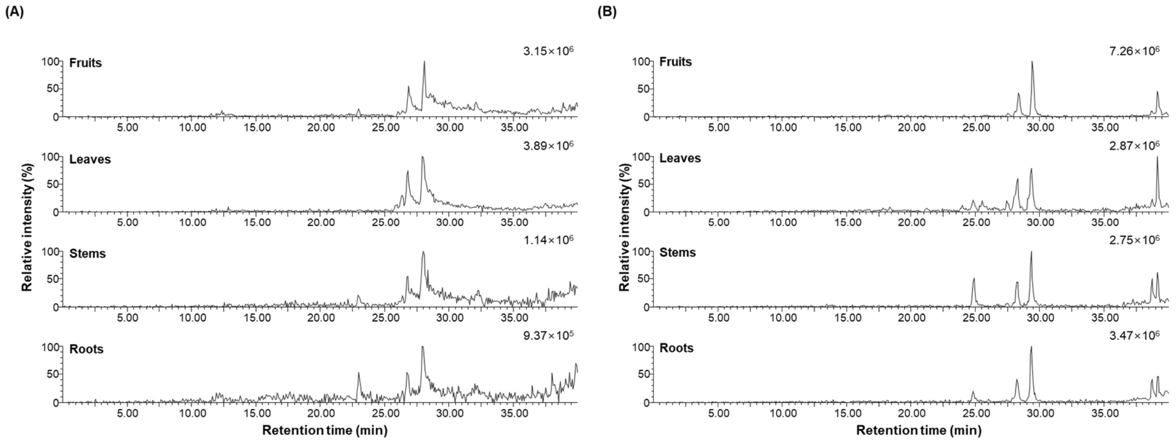

Figure S4. HPLC chromatograms of active compounds from eggplant extracts of different parts reacted with 2-nonenal. Extracts (10 mg/mL) from various parts of eggplant (fruits, leaves, stems, and roots) were incubated with 2-nonenal (10 mM) at 37°C for 72 h and then analyzed using HPLC. (A) Chromatogram showing a product with a molecular mass of *m*/*z* 493.5, formed by the reaction of *N*-*trans*-caffeoylputrescine (**1**) from eggplant extracts. (B) Chromatogram showing the product with a molecular mass of *m*/*z* 507.5, formed by the reaction of *N*-*trans*-feruloylputrescine (**3**) from eggplant extracts.

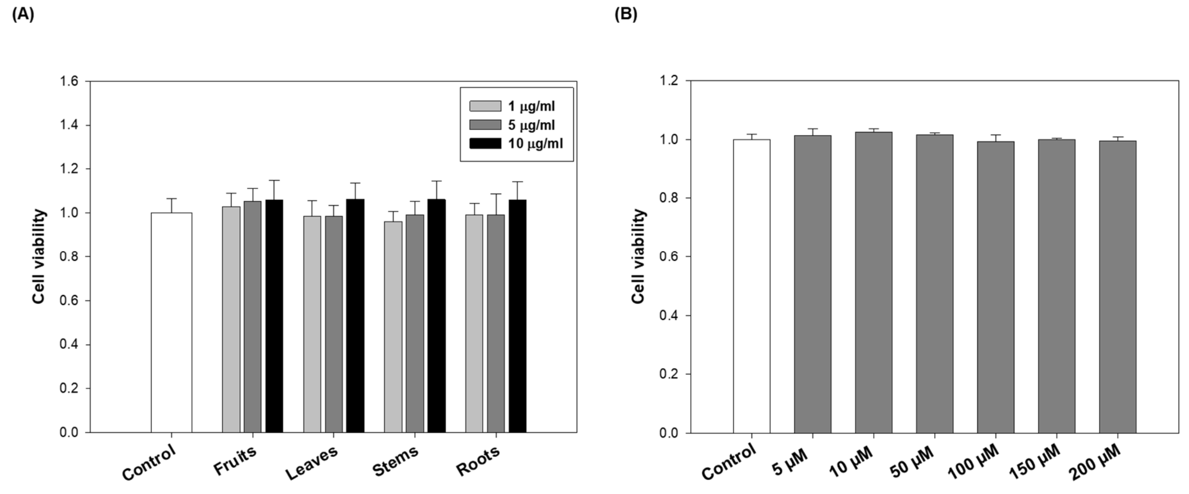

Figure S5. Cell viability of human keratinocytes treated with (A) extracts from various parts of eggplant or (B) *N*-*trans*-feruloylputrescine (**3**). Data is expressed as the mean ± SD of three independent experiments.
